# Preparation, Deformation Behavior and Irradiation Damage of Refractory Metal Single Crystals for Nuclear Applications: A Review

**DOI:** 10.3390/ma17143417

**Published:** 2024-07-10

**Authors:** Benqi Jiao, Weizhong Han, Wen Zhang, Zhongwu Hu, Jianfeng Li

**Affiliations:** 1 Northwest Institute for Nonferrous Metal Research, Xi’an 710016, China; jbenqi@163.com (B.J.); huzhongwu123@aliyun.com (Z.H.); jfli@c-nin.com (J.L.); 2 Center for Advancing Materials Performance from the Nanoscale, State Key Laboratory for Mechanical Behavior of Materials, Xi’an Jiaotong University, Xi’an 710049, China; wzhanxjtu@mail.xjtu.edu.cn

**Keywords:** refractory metal single crystals, preparation, deformation behavior, crystal orientation, irradiation damage

## Abstract

Refractory metal single crystals have been applied in key high-temperature structural components of advanced nuclear reactor power systems, due to their excellent high-temperature properties and outstanding compatibility with nuclear fuels. Although electron beam floating zone melting and plasma arc melting techniques can prepare large-size oriented refractory metals and their alloy single crystals, both have difficulty producing perfect defect-free single crystals because of the high-temperature gradient. The mechanical properties of refractory metal single crystals under different loads all exhibit strong temperature and crystal orientation dependence. Slip and twinning are the two basic deformation mechanisms of refractory metal single crystals, in which low temperatures or high strain rates are more likely to induce twinning. Recrystallization is always induced by the combined action of deformation and annealing, exhibiting a strong crystal orientation dependence. The irradiation hardening and neutron embrittlement appear after exposure to irradiation damage and degrade the material properties, attributed to vacancies, dislocation loops, precipitates, and other irradiation defects, hindering dislocation motion. This paper reviews the research progress of refractory metal single crystals from three aspects, preparation technology, deformation behavior, and irradiation damage, and highlights key directions for future research. Finally, future research directions are prospected to provide a reference for the design and development of refractory metal single crystals for nuclear applications.

## 1. Introduction

Refractory metals refer to metal materials with melting points higher than 2273 K, mainly including tungsten, molybdenum, tantalum, niobium, rhenium, and vanadium, as well as alloys based on these six metal elements. Refractory metals have an important position in cutting-edge fields such as aerospace, equipment manufacturing, and the nuclear industry, due to their excellent high-temperature performance, strong creep resistance, and high melting point [1,2,3,4,5]. Compared with traditional polycrystalline structural materials, refractory metal single crystals avoid high or low-temperature grain boundary damage, and no recrystallization ensures structural and property stability, thereby significantly improving component reliability, stability, and service life. Therefore, refractory metal single crystals are widely used in key fields such as aerospace, electronics, equipment manufacturing, and nuclear power [6,7,8]. Especially in the nuclear power field, many advanced energy systems require components that can maintain high-temperature operation with minimal deformation over long periods. For example, fuel elements used for direct heat-to-current conversion of thermionic systems must be able to withstand stresses greater than 10 MPa at temperatures up to 2000 K, with a service life exceeding 7 years [9]. The steady-state creep rate of these thermionic fuel elements must be kept to a minimum by using appropriate high-temperature structural materials. Refractory metal single crystals, due to the absence of grain boundaries and recrystallization, possess excellent high-temperature properties, especially creep resistance, and are therefore the preferred materials for high-temperature components of advanced nuclear reactors [10,11].

Due to the importance and uniqueness of refractory metal single crystals in cutting-edge fields, their preparation technology undoubtedly attracts special attention from scholars. However, due to the extremely high melting point and strong chemical activity in the liquid state of refractory metals, preparing high-quality refractory metal single crystals has always been a highly challenging topic. The Baikov Institute of Metallurgy and Materials Science, Russian Academy of Sciences (BIMMS RAS) has carried out abundant pioneering research works in the preparation technology of refractory metals since the 1960s. The electron beam floating zone melting (EBFZM) and plasma arc melting (PAM) techniques adopted can prepare large-size high-purity refractory metal single crystals, including tungsten, molybdenum, and their alloys [8,12,13,14]. In recent years, the BIMMS RAS has conducted research on the deformation processing of prepared large-size single crystals. For example, combining the EBFZM and rolling technology, Mo-3Re single-crystal foils 0.5 mm thick were processed, and it was indicated that these foils could be used to manufacture vacuum-sealed metal–glass parts in the future [15]. In addition, Liu et al. [9] also used EBFZM to prepare pure molybdenum, Mo-Hf, and Mo-Nb alloy single crystals and conducted a detailed systematic analysis for their high-temperature creep properties. Fujii et al. [16,17,18,19] used the secondary recrystallization method to prepare large-size molybdenum single-crystal plates with 2 × 40 × 180 mm and studied the rolling and recrystallization behavior of molybdenum single crystals. As is well known, perfect defect-free metal single crystals are difficult to prepare. It is difficult to completely suppress the formation of internal defects in the single crystals by the EBFZM and PAM techniques due to the high-temperature gradients. It is necessary to rely on relevant theoretical research and process innovation to continue optimizing and improving the size and structural integrity of single crystals. In the preparation of refractory metal single crystals, these two main preparation techniques have their own characteristics. Combining the advantages of EBFZM and PAM techniques is an important development direction for more efficient preparation of low-cost and high-quality refractory metal single crystals in the future.

In the field of basic theoretical research, refractory metal single crystals, with their advantages of high purity and structural integrity, have become irreplaceable materials for studying deformation behavior. For a long time, body-centered cubic (BCC) metals have exhibited unique deformation characteristics due to their special screw dislocation core structure, including slip asymmetry [20], anomalous slip [21,22], twinning anti-twinning asymmetry [23,24], and strong strain rate and temperature dependence of flow stress [25]. Due to the characteristics of their application and service environment, refractory metal single crystals are subjected to different forms of external loads, so the research focus for each type of single crystal varies, mainly including tensile [26,27,28], creep [29,30], fatigue [31,32,33], and impact [34,35,36]. Regardless of the load form, studying their deformation mechanisms has important reference significance for explaining their failure and breakdown causes. In terms of mechanical behavior, refractory metal single crystals have a complex deformation mechanism. It is necessary to consider the practical application and working environment of refractory metal single crystals to study their deformation behavior. The basic deformation mechanisms of refractory metal single crystals include slip and twinning, as well as coordinated deformation mechanisms [37,38,39,40]. In this paper, the corresponding mechanical properties of different refractory single crystals are introduced. Furthermore, the recrystallization behavior under the deformation–annealing effect is undoubtedly in need of great attention. The occurrence of recrystallization will rapidly deteriorate the creep properties of single crystals and even cause embrittlement [19,41]. The destruction of the single-crystal structure will pose a potential threat to the service safety of single-crystal devices.

The materials in nuclear reactors face extremely complex and harsh service conditions, including high temperature, fast neutron irradiation, high heat load, ion bombardment, etc. Under the above effects, various forms of defects will be introduced into the materials, including dislocation loops, dislocation networks, voids, bubbles, and precipitates [42,43,44,45]. As key high-temperature structural materials for nuclear reactors, refractory metal single crystals also suffer from harsh service conditions. The irradiation damage will lead to the irradiation hardening [46,47] and neutron embrittlement [48,49] of the materials, thereby destroying the microstructure and causing rapid degradation of mechanical properties, ultimately shortening the service life of components. Therefore, understanding the irradiation damage behavior of refractory metal single crystals and revealing the impact of irradiation damage on the microstructure and properties are of great significance for the safe and efficient operation of reactors. The combination of basic experiment and simulation is an important research method to study the irradiation damage of refractory single crystals, and is an important development direction for the characterization and evaluation of service behavior under extreme environments in the future.

As can be seen from the above, as the key structural materials for advanced power systems in the nuclear field, the preparation, deformation behavior, and irradiation damage of refractory metal single crystals will be a topic of great concern. In this review, we briefly overview the current research status of refractory metal single crystals in Section 1. The preparation methods of refractory metal single crystals are summarized in Section 2. The mechanical behavior and deformation mechanisms of refractory metal single crystals are reviewed in Section 3. Irradiation damage and its impact on microstructure and properties are outlined in Section 4. Finally, we discuss the future development directions of refractory metal single crystals.

## 2. Preparation of Refractory Metal Single Crystals

### 2.1. Development of Preparation Technology

The preparation of high-purity refractory metal single crystals is highly challenging due to the extremely high melting point and strong chemical activity in the liquid state, requiring the adoption of advanced methods that simultaneously possess purification processes and single-crystal growth. Currently, the preparation of high-purity refractory metal single crystals mainly adopts two main technical routes, including EBFZM and PAM. Table 1 shows a variety of refractory metal single crystals prepared by different techniques, most of which are used as heat-to-current conversion components in nuclear reactors.

Since the 1960s, the BIMMS RAS has been committed to the research and development of refractory metal single-crystal materials, and according to the service environment and design life of their key components, developed a series of refractory metal single-crystal materials. Molybdenum, tungsten, and other alloy single crystals were prepared by EBFZM and PAM, but there was no detailed report at that time. After decades of development, large-size tungsten and molybdenum alloy single-crystal rods (Φ50 × 300 mm), pipes (Φ(20~30) × 200 × 1 mm (wall thickness)), and plates (8 × 75 × 160 mm) [14,55] have been prepared. With the expansion of the application demand of single-crystal materials, and through improving the preparation method of single-crystal materials, great progress has been made in terms of the type and size of single crystals. Large-size refractory metal single-crystal bars, pipes, and cakes have been prepared by EBFZM [55]. Particularly, by using PAM, not only single-crystal bars with a diameter of more than 50 mm can be prepared, but also large-size pipes, sheets, and disks. In recent years, large-size single crystals have been deformed. For example, 0.5 mm thick Mo-3Re single-crystal foil was fabricated by warm rolling (1423 K) and cold rolling (673 K), which indicates that the foil can be used to make vacuum-sealed metal glass parts in the future [15]. In addition, since the 1990s, Zee et al. [7,9] have successively developed large refractory metal single-crystal materials including Mo, Mo-Nb, and Mo-Hf by using the EBFZM technique.

### 2.2. Electron Beam Floating Zone Melting

#### 2.2.1. Principles of the EBFZM Technique

EBFZM technology is a crucible-free melting technique, which is extremely important for the melting, research, and preparation of high-purity refractory metals [14]. Under vacuum conditions, an electron beam locally heats the rotating billet to form a molten zone. By controlling the shape and lifting of the molten zone and the rotation of the billet, high-purity refractory metal single crystals can be prepared. The EBFZM is used to purify refractory metals and alloys from gas and metal impurities and grow single crystals with expected dislocation structures.

As a crucible-free melting technique, EBFZM effectively avoids mutual contamination between the melt and crucible materials, enabling the preparation of high-purity refractory metal single crystals. Additionally, EBFZM can also better control the growth orientation and dislocation structure of single crystals. By optimizing process parameters, EBFZM technology can obtain large-size single crystals with low dislocation density and no low-angle grain boundaries. However, EBFZM technology also has some obvious shortcomings, such as the limited size and high vacuum environment requirements, which restrict the promotion in industrial applications.

#### 2.2.2. Refractory Metal Single Crystal Prepared by EBFZM

EBFZM technology is widely used to purify refractory metals and alloys from gas and metal impurities, as well as to grow single crystals with expected crystal orientations and dislocation structures. Parameters such as temperature gradient, growth rate, crystallographic orientation, and integrity of seed crystals during the crystallization process have a great influence on the structural defects and perfection of the final single crystals [8]. Otani et al. [58] used EBFZM to prepare refractory metal single crystals such as Mo, Ta, and W and grew single crystals 60 mm long and 10 mm in diameter after 3 h. To obtain larger-size single crystals with fewer defects, the improved EBFZM technology can be used to grow single-crystal rods (600–1000 mm) consistent with the seed crystal orientation [14]. In addition, tungsten single-crystal tubes can be grown in specially designed devices [13]. The growth axis is the <111> orientation, and there are several large sub-grains on the cross-section of the tube, separated by low-angle grain boundaries, with the crystal orientation deviation angle not exceeding 1–2°.

The dislocation structure of refractory metal single crystals grown by EBFZM depends on the temperature gradient and growth rate. Tungsten has the highest melting point among refractory metal single crystals, so growing high-purity “perfect” tungsten single crystals from melting by EBFZM is a formidable task. The grown W single crystals usually have a high dislocation density of around 10^−6^ cm^−2^, and these dislocations mostly aggregate into dislocation walls and networks, forming a characteristic dislocation substructure [12,59,60]. Therefore, considerable efforts have been made to improve the structural quality of W single crystals, which can achieve a dislocation density of 10^−5^ cm^−2^. In many cases, high-temperature annealing after the plastic deformation of single crystals can yield large grains of 20 mm, with the dislocation density reduced by two orders of magnitude and no low-angle grain boundaries [60]. To acquire the optimal conditions for recrystallization of W single crystals, Bozhko et al. [12] prepared high-quality tungsten single crystals with low dislocation density and no low-angle grain boundaries by combining controlled plastic deformation and recrystallization methods based on the EBFZM. The EBFZM is very useful for preparing high-purity metals and growing single crystals with determined orientations and the overall quality can be effectively improved by optimizing the process.

### 2.3. Plasma Arc Melting

#### 2.3.1. Principles of the PAM Technique

PAM is a new crucible-free melting technique developed by the BIMMS RAS. This technology uses a plasma heating device to heat the raw materials, which can quickly and uniformly melt refractory metals, thereby effectively avoiding mutual contamination between the melt and the crucible. The PAM method has a high-energy-density heating source, and the raw material specifications can be powder, plates, or rods. It can prepare high-purity single-crystal rods with a size exceeding Φ50 mm and a weight exceeding 10 kg, and large-size plates and tubes.

The prominent advantage of PAM technology is that it is not limited by the size and shape of single crystals. It can prepare oriented single crystals of various specifications, such as large-diameter rods, tubes, and plates, which greatly expands the application fields of refractory metal single crystals. In addition, PAM technology has relatively high energy utilization efficiency and relatively low processing costs. By optimizing the process, PAM technology is fully expected to achieve the goal of industrial mass production of large-size refractory metal single crystals. However, at the same time, due to the extremely high heating power of plasma arc melting, there are often more structural defects inside the single crystals. In addition, the complexity of the PAM equipment limits its promotion and application.

#### 2.3.2. Refractory Metal Single Crystals Prepared by PAM

**Figure 1:**
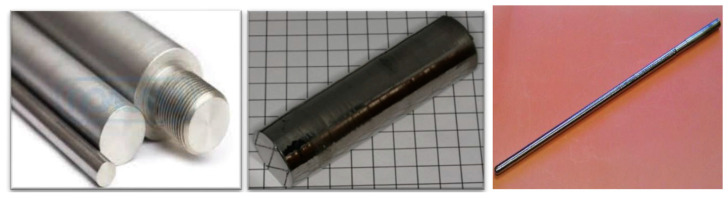
Tungsten monocrystalline rods of various shapes [57].

Compared with EBFZM, PAM technology has higher heating power and energy density, so it has unique advantages in preparing large-size refractory metal single crystals. Figure 1 shows the large-size oriented tungsten single crystal grown by PAM technology, about 800 mm [57,61]. To prepare refractory metal single crystals of specified sizes (such as tubular or hollow cones), the BIMMS RAS has achieved oriented growth of refractory metal single crystals of various sizes and shapes by developing the plasma generator scanning devices and auxiliary electromagnetic field control systems designed, achieving oriented growth of refractory metal single crystals such as molybdenum single-crystal rods with a diameter of 100 mm, Φ70 × 10 × L mm single-crystal tungsten tubes, 8 × 75 × 160 mm single-crystal tungsten laminates, and so on [55]. PAM technology can directly use powder as a raw material to prepare single crystals. The advantage of this technology is that it can reduce or avoid the preparation of polycrystalline original materials, such as sintering, stamping, and welding blanks, thereby greatly reducing the processing cost of single crystals. Skotnicova et al. [56] developed a method to directly use waste tungsten powder to prepare pure tungsten single crystals without the need for pre-melting alloys or pressing rods in an electric arc furnace. The large-size tungsten single crystals were prepared using a dual process combining plasma arc melting and electron beam melting.

A more economical and energy-saving method was developed, namely extracting directly from tungsten and molybdenum rods or wire materials of suitable alloying elements to prepare low-alloyed W-Mo, W-Nb, Mo-W, Mo-Nb, and Mo-Ta single crystals [62]. PAM technology avoids the complex process of preparing traditional polycrystalline original materials, significantly improving the preparation efficiency and economy of single crystals.

## 3. Deformation Behavior

### 3.1. Mechanical Properties

#### 3.1.1. Tensile—W Single Crystals

Tungsten single crystals, renowned for their excellent high-temperature performance, high recrystallization temperature, and compatibility with nuclear fuel, have become promising structural materials in the nuclear field, such as nuclear power devices, high-temperature fast reactors, international thermonuclear experiment reactor (ITER) metal reflectors, etc. [63]. Given the demanding conditions these materials face, it is imperative that tungsten single crystals exhibit both high tensile strength and ductility. Tensile properties are an important reference for studying and evaluating the service behavior of tungsten single crystals under such challenging environments. Table 2 shows the tensile property data of single-crystal and polycrystalline tungsten under different conditions.

**Figure 2:**
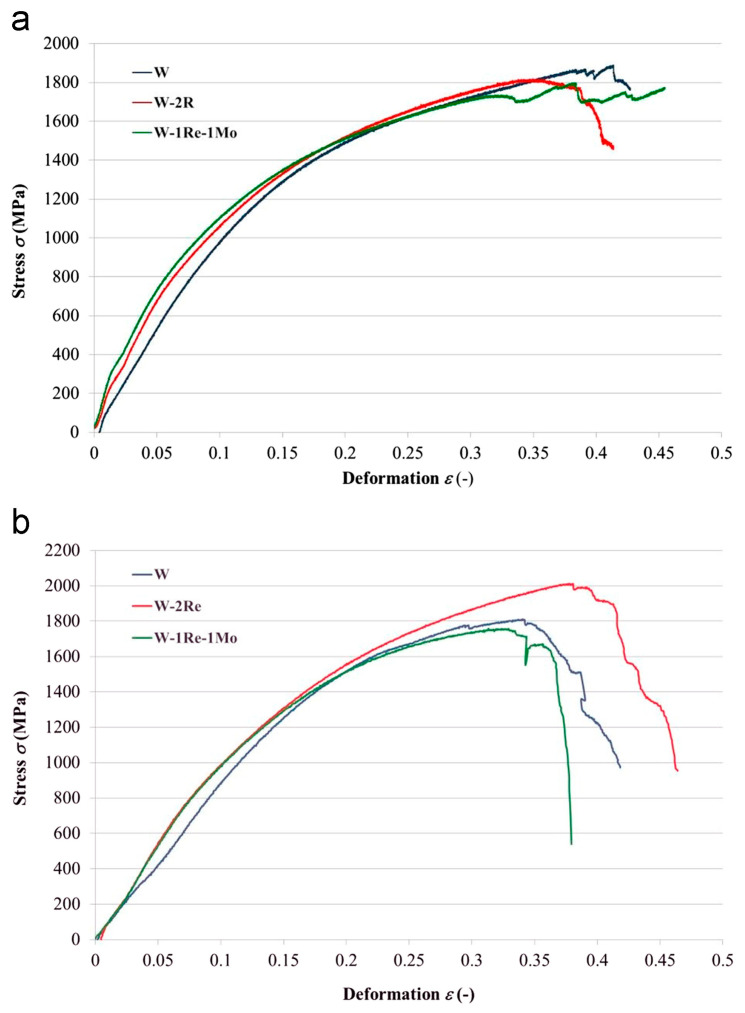
Work diagrams from pressure tests at various deformation rates for particular single-crystal specimens in relative stress–relative deformation ε [27]: (**a**) 0.2 mm/min and (**b**) 2 mm/min.

Table 2 illustrates significant variations in the tensile properties of tungsten, highlighting a pronounced dependence on crystal orientation and temperature [26,27,57]. Tungsten single crystals along the [001] orientation exhibit a gradual transition from elastic to plastic behavior with obvious strain hardening, while along the [011] orientation, they exhibit a higher and more pronounced yield point with lower strain hardening [26]. The stress–strain curve characteristics of tungsten single crystals vary greatly with crystal orientation, and only close to [110] does the orientation exhibit discontinuous yield behavior [28]. A comparative study between polycrystalline and single-crystal W-4Ta has revealed that the latter possesses superior high-temperature strength and plasticity, making it more suitable for use at high temperatures and high stresses [57]. It is worth noting that it can be concluded from Table 1 that the preparation methods of tungsten single crystals are EBFZM and PAM technologies, and the mechanical properties of single crystals prepared by these two technologies are reflected in Table 2. The tensile strength of the [001] W single crystals at room temperature is 760 MPa and 1812 MPa, and the elongation is 19.5% and 21%, respectively, which means that the mechanical properties of W single crystals prepared by PAM are better than those of EBFZM. Therefore, combined with the data in Table 1 and Table 2, it can be concluded that the mechanical properties of W single crystals are closely related to the preparation process, and the mechanical properties of PAM technology are superior to EBFZM. In addition, the alloy composition and deformation rate also have a significant impact on the tensile properties of tungsten single crystals. Figure 2 shows that there are obvious differences in the tensile deformation behavior of pure tungsten and its alloy single crystals, which is related to the specificity of the role of rhenium and molybdenum in the tungsten solid solution. When the deformation rate is reduced, the mechanical properties of W alloy single crystals decrease, but the ε*_r_* value remains at the same level [27].

The fracture behavior of tungsten single crystals depends on microcracks, pre-strain, and crystal orientation. Cleavage cracks can be introduced on the (010) plane by electrical discharge machining. At 77 K, the fracture stress is related to the crack length, the specimen becomes completely brittle, and the traces on the fracture surface depend on the stress level at the fracture. Moreover, as the temperature rises, the crack propagation mechanism changes from fast cleavage to slow cleavage, becoming “ductile cleavage” propagation at higher temperatures [64]. Although the {100} plane has the lowest surface energy, the {100} plane can act as a preferred cleavage plane, thereby preventing crack propagation [65]. In addition, the pre-strain also has a significant effect on the fracture behavior of tungsten single crystals. For instance, pre-stretching at room temperature enhances the brittle fracture stress of tungsten single crystals with [010] orientation containing microcracks at 77 K, and the fine river-like pattern may be caused by the dislocation multiplication mechanism acting at the crack tips [66]. The crystal orientation has a significant effect on the fracture behavior of single-crystal tungsten, especially on the fracture surface morphology. Relevant research shows that the {001} orientation is a brittle cleavage fracture, while tensile stress along the {011} orientation produces necking, which is a ductile fracture, and the fracture is in a “chiseled edge” shape [26]. Ma et al. [67] used molecular dynamics to analyze the influence of temperature and crystal orientation on crack propagation in tungsten single crystals under uniaxial load. The main deformation mechanism of cracks in the [001] direction is the slip bands, dislocations, and blunting, while in the [111] direction, cracks propagate in the form of blunting and voids.

#### 3.1.2. Creep—Mo Single Crystals

**Figure 3:**
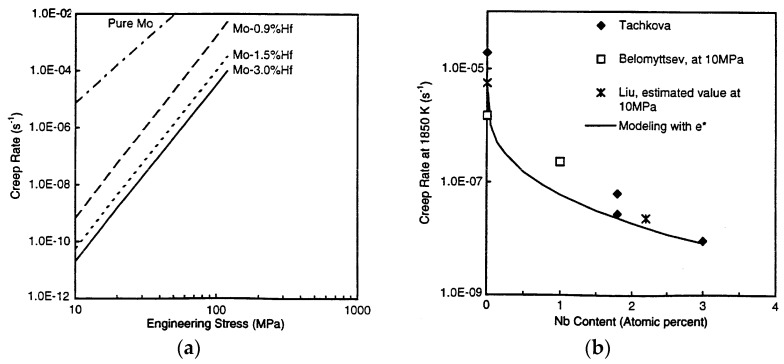
Creep properties of molybdenum and molybdenum alloy single crystals [9]. (**a**) Mo-Hf single crystal; (**b**) Mo-Nb single crystal.

The polycrystal materials of refractory metals are far from meeting the design requirements of nuclear components, due to their large creep rate at high temperatures. In contrast, the corresponding single-crystal materials do not experience grain boundary sliding during high-temperature deformation, and the microstructure is stable, exhibiting excellent high-temperature creep resistance. There are relatively few reports on the creep behavior of refractory metal single crystals because of the long duration and ultra-high-temperature conditions, with relevant reports only focusing on the creep properties of molybdenum single crystals. Figure 3 shows the creep properties of molybdenum and molybdenum alloy single crystals [9].

The main slip system of the [011]-oriented molybdenum single crystals under creep at (0.5–0.76) Tm is {112} <111> [30]. This process is characterized by an uneven distribution of slip, with activity predominantly concentrated in narrow, regularly spaced bands. The X-ray diffraction results show that the dislocation cell boundaries are formed by inclined walls of equidistant dislocations [68]. The above research results suggest that the creep mechanism of molybdenum single crystals is closely related to the evolution mechanism of dislocations and substructures. However, the evolution process of dislocations and substructures is influenced by many factors, mainly including temperature, stress, pre-deformation, solute types and distribution, etc. Dekhtyar et al. [30,69,70] conducted continuous research on the dislocation evolution mechanism during the creep process of molybdenum single crystals for several decades, believing that the dislocation evolution mechanism is closely related to the strength of the applied stress and the degree of pre-deformation.

**Figure 4:**
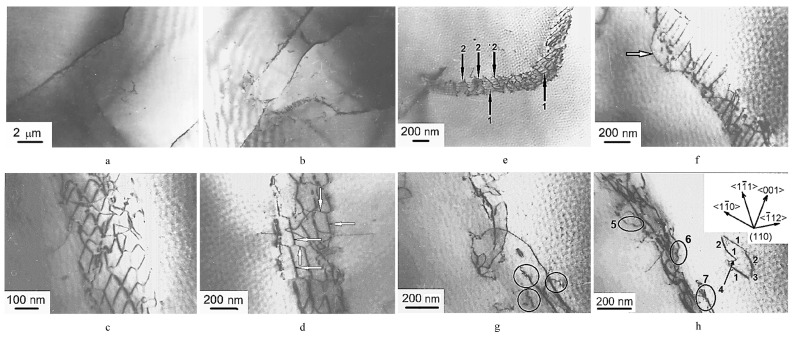
Dislocation substructure of bent Mo single crystal after creep for 1000 h in the zones where intense slip was not observed [70]: (**a**,**b**) general view of subgrains; (**c**) irregular tetragonal net; (**d**) irregular hexagonal net with screw (some of them are indicated by horizontal arrows) and edge (some of them are indicated by vertical arrows) components; (**e**) subboundary consisting of mixed dislocations (arrow 1) entwined with a helicoidal dislocation (arrow 2) formed from a braided component of dislocation net; (**f**) a dislocation wall formed by parallel mixed dislocations fixed by dislocation (indicated by arrow) close to screw orientation with numerous helicoid components; (**g**) a subboundary with dislocation loops and helicoids (indicated by ellipsoids); (**h**) a subboundary with dislocation loops, and separate loop comprised of segments of sessile edge dislocations (1), segments of screw dislocations (2) of one net system, and segments of glissile edge dislocations (3, 4) of other net system. The helicoids are indicated by ellipsoids 5, 6 and 7. The crystallographic directions indicated in this figure are the same for the entire Figure 4.

Under strong stress at 1633 K, the creep rate of molybdenum single crystals is controlled by the Weertman mechanism, which involves edge dislocation climbing out of the sub-grain boundaries, while the creep rate is controlled by the diffusion recovery mechanism within the sub-grain boundaries under weak stress [30]. When low stress is applied, edge dislocations tend to accumulate in the form of sub-grain boundaries. When the external stress is sufficient for dislocations to overcome the mutual repulsion, the edge dislocations and mixed dislocations intersect with each other and relax after forming a certain concentration, realizing a creep mechanism controlled by dislocation climbing [69]. Recently, Dekhtyar et al. [70] found that the creep rate of pre-bent single crystals was 4–5 orders of magnitude lower than that of un-strengthened single crystals within 1000 h in the study of the internal pressure creep resistance of molybdenum single-crystal tube/shells. It was believed that the edge component of the dislocation was exhausted and disappeared in the creep test, and the cross-slip of the screw dislocation formed a large number of dislocation rings and spirals under the action of bending. These dislocation rings and helices are transformed during the creep process, and the dislocation creep gradually changes to diffusion. It can be seen from Figure 4c–g that the polygonal irregular mesh-like quadrilaterals and hexagons gradually transform into dislocation loops and spirals.

In addition, the type and distribution of solutes also have an important influence on the creep behavior of molybdenum single crystals. The addition of Zr and C can maximize the improvement of the creep properties of molybdenum single crystals. This improvement is attributed to the interactions between the mobile dislocations and the micro-segregations caused by the introduction of a small number of additives [71]. Tachkova et al. [72] found that doping up to 11% niobium into molybdenum single crystals can improve their creep resistance and believed that when the alloy addition amount is 1%, the creep rate can be reduced by about 10 times. The creep properties of molybdenum-based alloy single crystals at high temperatures of 0.6 Tm are closely related to the type of solid solution atoms. The size mismatch between the matrix atoms and solute atoms is the fundamental driving force, which improves the high-temperature creep resistance of materials. Dislocation climbing and viscous resistance are two competing processes during creep. Mo-Hf single crystals exhibit superior strengthening effects compared to Mo-Nb single crystals. This is due to the larger solute–solvent atomic size mismatch in Mo-Hf single crystals than in Mo-Nb, which contributes to their enhanced creep resistance [7,9]. Zhang [11] found that with an increase in solute atom niobium content, the internal pressure creep resistance of Mo-Nb alloy single crystals was enhanced, and the steady-state creep rate and sensitivity to stress were reduced.

#### 3.1.3. Fatigue—Nb Single Crystals

Fatigue is a form of load that Nb single crystals often encounter during service. The slip deformation type and fatigue life of Nb single crystals during torsional fatigue are related to the cyclic strain amplitude and interstitial impurity content. Interstitial impurities play a crucial role in altering the slip mechanism by suppressing the cross-slip of dislocations [73]. The fatigue asymmetric deformation in Nb single crystals can lead to local rapid deformation and fatigue failure of the material, which can be explained by the dislocation mechanism involving asymmetric slip and repeated cross-slip on the {112} and {123} planes [74]. Nb single crystals, with their favorable orientation for single slip deformation, exhibit distinct characteristics under cyclic deformation. Under cyclic deformation with a wide range of plastic strain amplitudes and cumulative strain ranges at 400 K, they exhibit a shape change caused by asymmetric slip [75]. Anglada et al. conducted a thorough investigation into the asymmetric behavior of fatigue deformation in Nb single crystals, considering factors such as deformation temperature, strain rate, and crystal orientation [31,32,33,76]. In their study, they initially focused on the phenomena of cyclic hardening, changes in crystal shape, and the asymmetry of hysteresis loops. They proposed that the cyclic stress–strain curve of Nb single crystals can be divided into four distinct hardening regions. As the temperature decreases or the strain rate increases, the stress asymmetry in the saturation stress increases. The stress asymmetry is consistent with the asymmetric slip characteristics of screw dislocations, and evidence of the influence of the positive stress component on the critical resolved shear stress (CRSS) plane slip was obtained [31]. Subsequently, they also found that cyclic hardening strongly depends on crystal orientation [32]. It has been discussed that the cyclic flow stress of two Nb single crystals with different axial directions exhibits a dependence on temperature and strain rate, and it was believed that this dependence is independent of crystal orientation [34]. In addition, when cyclic deformation reaches saturation, the Bauschinger effect of niobium single crystals at small strain amplitudes and different temperatures was also studied. By improving the method proposed by Cottrell, the predicted average internal stress values of the matrix agree well with the experimentally measured values [76]. Lin et al. [77] studied the cyclic hardening and stress asymmetry of Nb single crystals and the hardening curve can be divided into three stages. The dislocation configuration in the first stage is mainly characterized by a high-density network and debris ring, the formation of a dislocation bundle in the rapid hardening stage, and the fully developed dislocation bundle structure in the saturation stage.

#### 3.1.4. Impact—Ta Single Crystals

**Figure 5:**
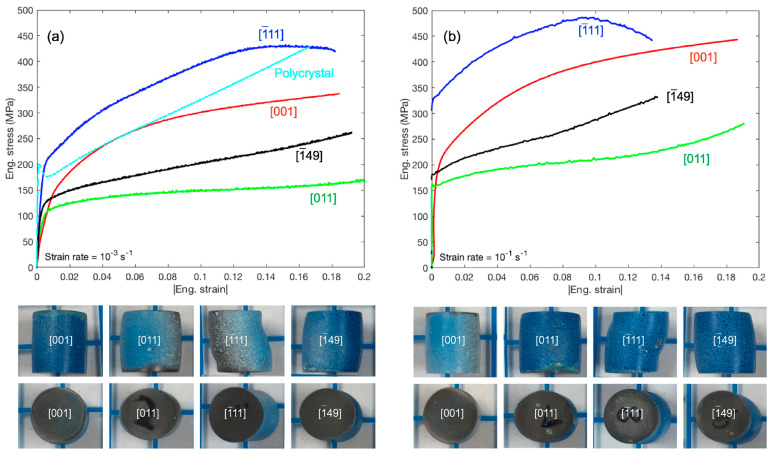
Stress–strain responses of four single crystals and polycrystalline tantalum upon quasi-static compression at strain rates of (**a**) 10^−3^ s^−1^ and (**b**) 10^−1^ s^−1^. Lower figures show side and bottom profiles of deformed single crystals [78].

Refractory metal single-crystal components may be subjected to high-speed impact loads during service, such as debris from aircraft, meteorites, etc. Therefore, studying the deformation behavior under impact loads is crucial for assessing the impact of damage resistance and optimizing protective designs. The deformation mechanisms of metals are inherently complex, particularly under dynamic conditions, which poses challenges for ensuring high reliability in their applications [34]. Twinning is a typical deformation mechanism at high strain rates. The {112} twins are widely present in tantalum single crystals during impact, and the main formation mechanism is the action of transverse release waves. The density distribution of twins in the single crystal is uneven and more concentrated in the sample edge region. It can be seen from Figure 5 that the initial crystal orientation of the four Ta single crystals has an impact on the initial yield and strain hardening behavior [78]. Increasing the strain rate by two orders of magnitude improves the overall flow strength, but maintains a very similar orientation dependence. The mechanism of shock wave- and release wave-induced twinning was analyzed by crystal plasticity simulation. It is believed that when the loading direction is in the [011] or [111] orientation, twinning is the main deformation mechanism. Under Taylor impact loading, tantalum single crystals are dominated by dislocation slip in the {112} plane, and twinning/anti-twinning in the {112} plane has a significant anisotropic effect on slip resistance [79,80]. In addition to twinning, the deformation of tantalum single crystals under impact loads is also accompanied by a large number of dislocation slips. Therefore, accurately describing the impact of tantalum single crystals requires consideration of the anisotropic effect of the twinning/anti-twinning in the {112} plane on slip resistance [35]. Compared with polycrystalline samples, tantalum single crystals exhibit unusually high deformation localization and significant plastic anisotropy, showing strong axial symmetry, and this symmetry varies with different crystal orientations [34]. Experiments explored the role of single-crystal orientation in Taylor impact tests for the first time, clearly demonstrating the importance of crystal orientations in high-strain-rate deformation.

### 3.2. Deformation Mechanism

#### 3.2.1. Dislocation Slip

The plastic deformation of typical metals and alloys is controlled by the collective motion of dislocations along specific slip planes and directions. The slip planes of body-centered cubic refractory metals are not very stable due to the lack of close-packed planes, and there are currently controversies [37].

*W single crystals*—Compared with molybdenum, niobium, and tantalum single crystals, the slip trace analysis of W is relatively difficult to acquire due to a very high melting point (3683 K). When the strain of tungsten single crystals reaches above 10%, slip traces can be identified on the surface [81]. Kaun et al. [82] conducted a comprehensive study on the slip traces in tungsten single crystals with various orientations, including [211], [321], [110], and [491] orientations, using both optical and electron microscopy. They found that {110} planes were the main slip planes, and weak sets of {112} and {123} slip planes were also observed in [491]- and [110]-oriented single crystals. Tabata et al. [83] performed in situ tensile tests on [100]- and [110]-oriented tungsten single-crystal foils at room temperature, but only observed sets of {112} planes. Therefore, the slip planes in tungsten single crystals are quite inconsistent.

**Figure 6:**
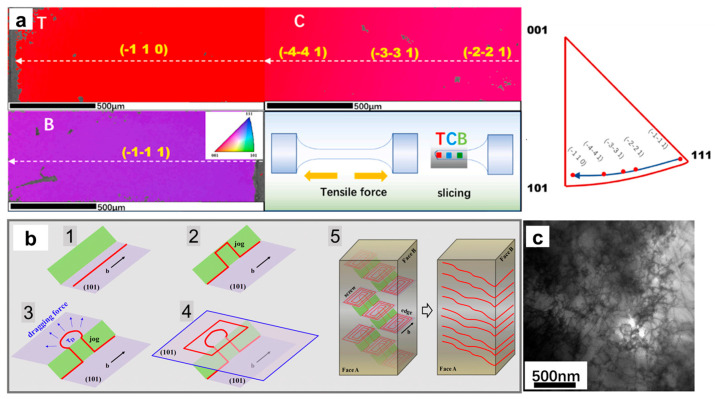
Sliding deformation mechanism of the <111>-oriented Mo alloy single crystal. (**a**) Schematic diagram of crystal orientation evolution IPF and lattice rotation during the tensile process [87]; (**b**) double cross-slip mechanism of screw dislocation [85]; (**c**) TEM characterization of screw dislocation distribution [86].

*Mo single crystals*—The slip planes of molybdenum single crystals are sets of {110} planes when tensile tested at 4.2 K, but the slip traces at 77 K cannot be determined using optical microscopy [24]. Kaun et al. [82] mainly observed sets of {110}-plane slip in molybdenum single crystals during room-temperature tensile experiments. Vesely et al. [38] believed that when pure molybdenum single crystals undergo room-temperature tensile deformation, slip occurs more easily on sets of {110} planes than on {123}, {112}, or higher-index planes. Slip bands are only observed on sets of {110} planes above 1573 K in molybdenum single crystals. The slip planes are easier to determine at high temperatures because mixed dislocations usually have determinable slip planes [84]. Our previous work proved that the slip planes of [111]-oriented Mo-3Nb single crystals at room temperature are sets of {110} planes, controlled by the double cross-slip multiplication mechanism of screw dislocations, as shown in Figure 6b,c [85,86]. The latest work shows that the lattice rotates selectively from the initial (-1-11) to (-110) when Mo alloy single crystals undergo tensile stress along the <111> orientation, as shown in Figure 6a [87].

*Nb single crystals*—The main slip planes of niobium single crystals appear on {110} planes when deformed below 175 K. There are also some exceptions. For example, the slip planes are {112} planes below 175 K, but slip traces can be observed on both {110} and {112} planes above 175 K. It is believed that the slip planes depend on temperature, loading direction, and single-crystal orientation [88]. In addition, for Nb single crystals with orientations near the [111]-[110] boundary of the stereographic triangle, the slip planes are {112} planes at 77 K [89].

*Ta single crystals*—The slip planes of tantalum single crystals show contradictory research results. Mitchell et al. [90] found that the slip traces at 4.2 K are the maximum resolved shear stress plane (MRSSP) in tensile-deformed tantalum single crystals. However, Shields et al. [91] demonstrated that {110}-plane slip occurred in tensile samples of tantalum single crystals at 4.2 K, while sets of {112}-plane twinning occurred in compression samples. Wasserbäch et al. [21,92] found that anomalous slip occurred in tantalum single crystals at and below 77 K, which occurred on the {110} plane. These results indicate that slip also occurs on the {110} plane in tantalum single crystals, but the MRSSP has more characteristics of a macroscopic response.

From the slip behavior of single crystals of these common refractory metals W, Mo, Ta, and Nb, it can be concluded that there are no consistent slip planes in BCC metal single crystals. The direct experimental observations of slip traces indicate that there may be multiple competing slip systems, and the activation ability of each slip system depends on many factors, including materials, temperatures, purity, and loads. Generally speaking, the slip takes the form of planes and almost always occurs on the sets of {110} planes at low temperatures. As the temperature rises, the diffuse wavy slip observed on the sets of {110}, {112}, and {123} planes (in order of increasing rarity of slip systems) gradually increases, eventually approaching pure slip on the MRSSP.

Anomalous slip, a deviation from Schmid’s law, is a notable behavior in BCC metals within limited temperature ranges and certain stress axis crystal orientations, resulting in a large amount of plastic slip on the sets of {110} planes with low shear stress. At 77 K, the deformation of high-purity Nb single crystals mainly occurs in the low-stress system (0$\overset{-}{1}$1)[$\overset{-}{1}$11] and (0$\overset{-}{1}$1)[111], and slip rarely occurs on the main system ($\overset{-}{1}$01)[111] and conjugate system (101)[$\overset{-}{1}$11]; that is, the (0$\overset{-}{1}$1) slip plane dominates [93]. The Mo-5Nb and Mo-5Re single crystals exhibit anomalous slip during compression deformation at 77 K. For Mo-5Nb single crystals, the contribution of anomalous slip to overall slip is much greater, but in Mo-5Re alloys, this contribution is not very large [94]. However, the origin of the anomalous slip remains unresolved. Hsiung et al. [22] used TEM to study the abnormal slip behavior of dislocation substructures in Mo single crystals when compressed to 0.4% total strain at room temperature and found that the abnormal slip of the (0$\overset{-}{1}$1) plane was caused by the mutual capture of a_0_/2[111] and a_0_/2[1$\overset{-}{1}$1] coplanar dislocation arrays on the ($\overset{-}{1}$01) main slip plane. In addition, in situ micro-pillar compression experiments and discrete dislocation dynamics simulations have revealed significant anomalous slip in tungsten single crystals, attributed to cross-kinking. The tungsten single crystal has an abnormal (0$\overset{-}{1}$1) slip plane under micro-pillar compression [95].

#### 3.2.2. Deformed Twinning

At lower temperatures or under high-strain-rate deformation, twinning is often more likely to occur in many metals. At room temperature and slower strain rates, the plastic deformation of face-centered cubic (FCC) metals with low stacking fault energy usually produces twinning. However, BCC metals, due to their high stacking fault energy, do not easily produce deformation twinning. Therefore, dislocation slip usually dominates the plastic deformation in BCC metals, while deformation twinning is only activated under extreme conditions such as high-speed impact and low temperatures. The twinning mechanism of BCC refractory metal single crystals has attracted extensive attention in recent years.

At high strain rates, for example, under impact loading, the applied stress rate exceeds the plastic relaxation produced by dislocation nucleation and multiplication. Pang et al. [80] found that (112), (121), and (211) twinning was activated during the 6 GPa plate transverse release shock experiment in Ta single crystals, and the reason for the formation of twinning was the high shear stress generated by the inclination of the lateral release wave. Subsequently, the research group also found that the defect evolution and response of Ta single crystals with different orientations are highly dependent on the orientation and their position in the sample. To further investigate the effect of crystal orientation on twinning, one-dimensional planar shock waves were used to impact Ta single crystals along the [001], [011], and [111] orientations. It is believed that the shear twinning is the main deformation mechanism at the shock front of single crystals subjected to loads of 15.4 GPa and 21.7 GPa, contrasting with samples loaded at 6.4 GPa, where this mechanism is less prominent [96]. In addition to plane impact-induced twinning, Chiem et al. [97] used a split Hopkinson bar to study the plastic deformation behavior of [110]- and [111]-oriented high-purity tungsten single crystals at room temperature. They discovered that twinning occurs within a dynamic strain rate regime, with an increase in twin density correlating positively with the strain rate.

There are many unusual aspects of the mechanical behavior of metal single crystals at low temperatures, and twinning is generally also observed at ultra-low temperatures where the dislocation migration rate is slower. Boucher et al. [98] studied the effect of different dislocation distributions on twinning behavior and believed that a uniform distribution of dislocations is the main condition for effectively suppressing twinning. Shields et al. [91] found that (011) slip is the main mode for high-purity Ta single crystals under tensile deformation at 4.2 K, while compressive deformation is dominated by twinning, and slip near the [001] orientation is only observed in a very small range. Ta single crystals exhibit high-rate continuous strain softening under primary shear, exhibiting anomalous and highly irregular deformation twinning morphologies, including typical straight plates and thin plates [40].

In addition, unstable twinning has also been found in BCC metals deformed at room temperature and slow strain rates. Most deformation twins in BCC metals are unstable, and spontaneous detwinning occurs after unloading by in situ transmission electron microscopy at atomic scale at room temperature and low strain rate [99]. The instability of BCC twinning is closely related to inclined twinning boundaries which provide the driving force for spontaneous twinning.

#### 3.2.3. Slip and Twinning Coordinate Deformation

The main plastic deformation mechanisms of crystalline materials are dislocation motion and shear twinning, which compete to control the plastic response of metals and alloys. The competition between these two deformation mechanisms is controlled by many factors, including crystal orientation, strain rate, deformation temperature, loading mode, and grain size. To study the competition mechanism between dislocation motion and shear twinning under the above factors, researchers have conducted extensive studies on the deformation behavior of refractory metal single crystals under different experimental conditions.

*Crystal orientation*—The deformation mechanism of Nb single crystals exhibits a strong crystal orientation dependence, in which the slip is the main mechanism deformed near the [001] axis direction, while the deformation mechanism of compressed samples along the central triangle direction and near the [011]-[111] edge is mainly twinning [39].

*Strain rate*—Dislocation slip and twinning are competitive under a high strain rate in Ta single crystals. The proportion of twins in all orientations is very low and the [110]-oriented crystal has the highest amount of twinning when the peak normal stress is 25 GPa [100].

*Deformation temperature*—To study the transition process from deformation twinning to dislocation slip, molecular dynamics simulation results show that the deformation mechanism of Ta single crystals transitions from twinning to slip, and this transition is accompanied by an increase in temperature and rapid stress relaxation [101].

*Loading mode*—Deformation twins were introduced in Ta single crystals under high strain rates (10^4^ s^−1^) through dominant shear and uniaxial compression [102]. The mechanical and microstructural responses exhibit different behaviors under shear and compression loads, with the shear-dominated deformation mechanism being more conducive to the transition between slip and twinning.

Grain size—In small-sized BCC single crystals, large surface areas can easily make bulk dislocation sources unstable. Therefore, dislocations and twins nucleating from the surface become a competitive deformation at room temperature and low strain rate. The competition between twinning and dislocation slip can be regulated by changing the loading orientation, which is attributed to the competitive nucleation mechanism of defects in nanoscale BCC crystals [103]. The competition between the dislocation slip and deformation twinning in BCC nanocrystals is strongly influenced by the deformation conditions. Under uniaxial tensile conditions, dislocation slip is the main deformation mechanism, while shear twinning is the main deformation mechanism under non-uniaxial stress conditions [104]. These results reveal the deformation mechanisms of BCC nanocrystals under complex loading conditions and provide new insights for understanding the plastic deformation mechanisms of BCC metals and alloys.

### 3.3. Deformation–Annealing Induced Recrystallization

**Figure 7:**
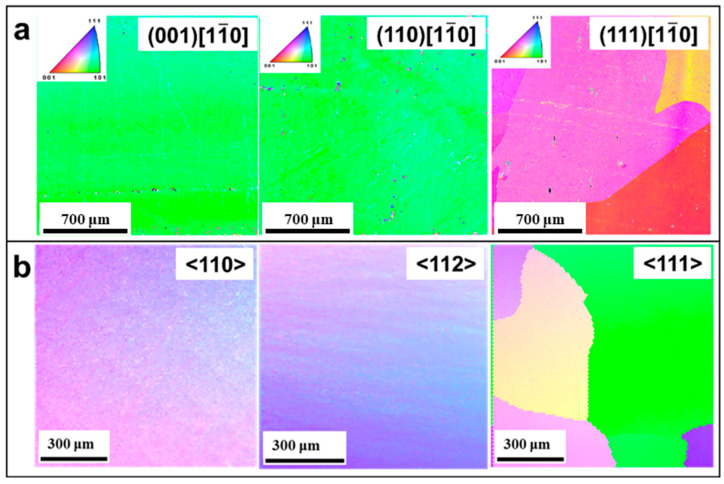
(**a**) Recrystallization occurred after annealing of Nb single crystals after rolling [41]; (**b**) recrystallization occurred after annealing of Mo single crystals after cold compression [108].

The combination of plastic deformation and annealing is usually an effective method to regulate the microstructure of materials and improve the processing performance of single crystals. As is well known, under appropriate annealing conditions, recrystallization often occurs, which will destroy the structural stability. The damage to the single-crystal structure can cause great trouble in actual production and even pose a potential threat to the service safety of single-crystal devices. The recrystallization behavior of BCC metal single crystals was first discovered in Fe-Si alloy single crystals [105]. In subsequent studies, it was found that recrystallization occurred at lower annealing temperatures in the {111}<112>-oriented Fe-3%Si single crystals after rolling, while the {110}-oriented single crystals after rolling were the most difficult to recrystallize [106,107]. This interesting recrystallization behavior is called the “orientation effect”, and it has also been observed in Nb and Mo single crystals [41,108,109], as shown in Figure 7.

Recrystallization usually occurs in the center of Mo and Ta single crystals, rather than at the edges, when subjected to a rolling reduction of 60% and annealed at 1473–1773 K for 1 h [17]. As the rolling temperature increases, the sharpness of the rolling texture of Ta single crystals decreases strongly, while the texture sharpness of Mo decreases slightly. Srinivasan et al. [41] studied the static recrystallization behavior of Nb single crystals, and the results showed that no recrystallization occurred in rolled samples in the (001) and (110) planes, while partial recrystallization occurred in rolled samples in the (111) plane after annealing at 1000 °C and 1200 °C. The above research results show that the static recrystallization behavior of single crystals has a very strong orientation dependence, so the nucleation problem of recrystallization must be clarified. The difference in recrystallization between single crystal and polycrystalline is that single crystals are not affected by grain boundaries, and the appearance of new grains can be easily observed in a single crystal. The orientation of new grains and their boundaries can be easily determined. However, there has been much controversy about the nucleation mechanism of recrystallization for single crystals. Hu et al. [110] believed that the merging of adjacent sub-grains through the dissolution or annihilation of their mutual boundaries is the nucleation mechanism. On the other hand, Walter et al. [111] pointed out that simple sub-grain boundary migration is a more likely mechanism. Therefore, to comprehend the recrystallization behavior of single crystals, it is essential to first comprehensively and accurately describe the cold-worked state under various orientations, taking into account the substructure’s shape, size, and misorientation. In studying the recrystallization behavior of Ta single crystals, Vandermeer et al. [112] found that a dislocation cell structure was only formed in the (111)[1$\overset{-}{1}$0] orientation during rolling, and believed that wide cell size distribution, rapid sub-grain growth, and high lattice curvature were the main reasons for easy recrystallization.

## 4. Irradiation Damage

### 4.1. Irradiation Damage in Refractory Metal Single Crystals

The irradiation effect of materials refers to the interaction between incident particles and lattice atoms of materials, which includes the processing of collision, defect formation, and microstructure evolution [113]. Such changes in the microstructure of materials caused by high-energy particles are called irradiation damage. It may cause material swelling, hardening, embrittlement, and other phenomena, which lead to a decrease in material toughness, increased brittleness, increased ductile–brittle transition temperature, and degradation of the physical, mechanical, and chemical properties of materials, and affects the service life of reactor materials [114].

Refractory metal single crystals have broad application prospects in key components such as the first wall and divertor of fusion reactors, which is attributed to their excellent high-temperature mechanical properties and irradiation resistance. However, the harsh service environment of fusion reactors places extremely high demands on materials, such as fast neutron irradiation, high heat loads, ion bombardment, etc., which can trigger complex irradiation damage processes in refractory metal single crystals, producing displacement cascades, defect clusters, precipitates, helium bubbles, etc., leading to material swelling, hardening, embrittlement, ductile–brittle transition, and other performance degradation, ultimately shortening the life of components [3]. Over the past half-century, scholars have carried out experimental and theoretical studies on the irradiation effects of refractory metal single crystals, achieving fruitful results and greatly advancing the understanding of the irradiation damage mechanisms of materials.

### 4.2. Irradiation Hardening

**Figure 8:**
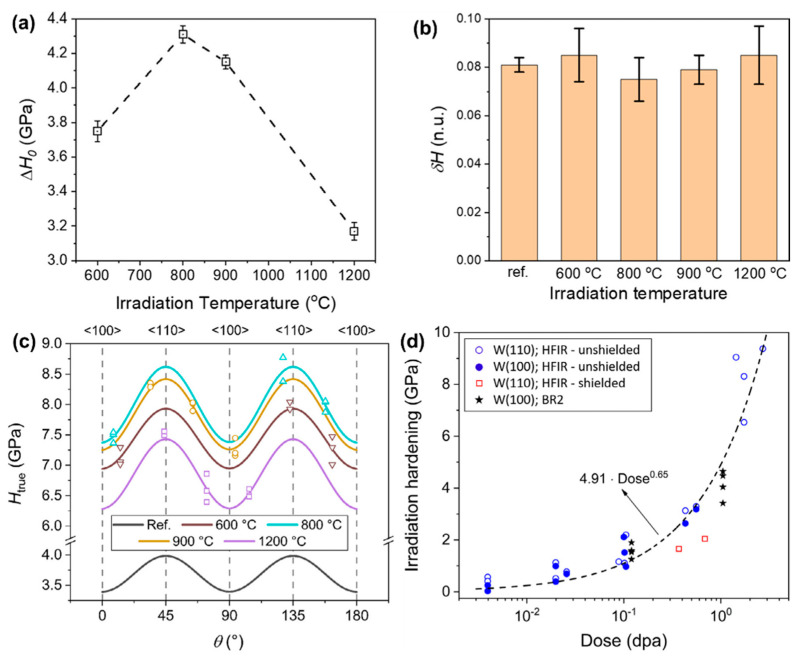
(**a**) Evolution of irradiation hardening, ∆H_0_ = $H_{0}^{irr}-H_{0}^{ref}$ with irradiation temperature; (**b**) relative amplitude, δH, obtained for the different samples; (**c**) evolution of H_true_ with rotation angle θ for different irradiation temperatures; (**d**) irradiation hardening, expressed as a difference in the hardness measured before and after the irradiation, of single-crystal W after neutron irradiation in different reactors under various conditions with accumulated dose [47].

Irradiation hardening is a common phenomenon in refractory metal single crystals under irradiation environments. It is manifested as an increase in material yield strength and hardness, and a decrease in plasticity and toughness. The high-density point defects, dislocation ring, and precipitation induced by irradiation can hinder the dislocation movement, which is the main mechanism leading to hardening. Scholars have carried out numerous experimental studies on irradiation hardening. As early as 1977, Takamura et al. [115] found that low-temperature neutron irradiation significantly increased the yield stress of Nb single crystals, believing that this was due to the pinning of dislocation motion by irradiation-induced point defects. Nagakawa et al. [116] found that the yield stress of Nb single crystals increases in proportion to the square root of the irradiation dose. Therefore, it can be concluded from the above analysis that the yield stress of Nb single crystals after irradiation depends heavily on the irradiation dose, and the yield stress increases with an increase in irradiation dose. In particular, the yield stress of Nb single crystals increases in proportion to the square root of the irradiation dose. On this basis, they proposed a solid solution strengthening model, believing that interstitial atom defects produced by low-temperature irradiation at 15 K can hinder the propagation of kinks, thereby causing the material hardening. Aono et al. [117] found that the annealing behavior of irradiated molybdenum single crystals exhibited a change from irradiation softening to irradiation hardening, corresponding to the migration of vacancies near 500 K. Yin et al. [47] systematically characterized the significant anisotropy in the microhardness of W single crystals with different crystal planes after fast neutron irradiation with 1 dpa. Figure 8 shows that irradiation hardening reaches its maximum at 1073 K, and the δ_H_ after irradiation remains almost constant compared to the reference value, independent of the irradiation temperature. The [110] orientations at 45° and 135° lead to maximum hardening values at all temperatures, all data conform to the same trend described by the typical power law, and no saturation of hardening is observed at least below 3 dpa, as shown in Figure 8c.

### 4.3. Irradiation Embrittlement

**Figure 9:**
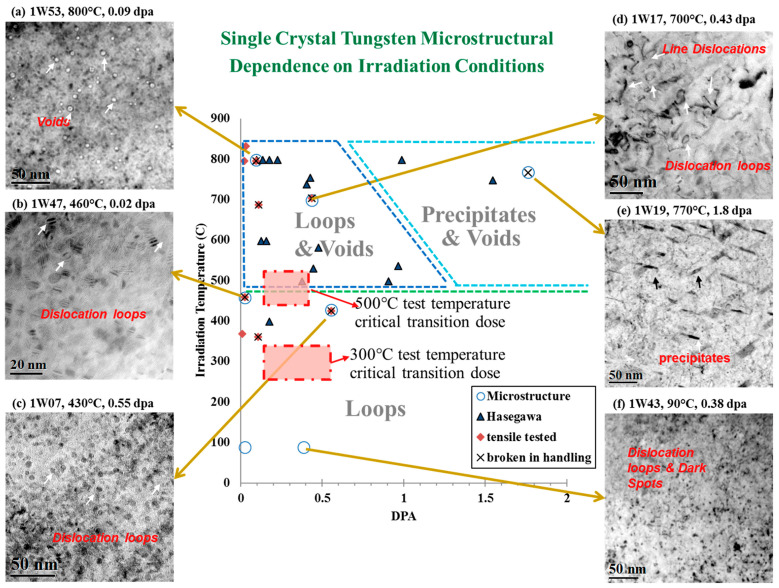
Summary of microstructure from the TITAN/PHENIX program [119].

In addition to the hardening effect, irradiation can also cause severe embrittlement of refractory metal single crystals, manifested as a sharp decrease in plasticity and toughness, and a transition in fracture form from ductility to brittleness. It is mainly attributed to the high-density dislocation loops, helium bubbles, precipitates, etc., induced by irradiation, which promotes the nucleation of microcracks, accelerates crack propagation, and reduces the fracture toughness of materials. After being subjected to fast neutron irradiation, Mo single crystals become brittle fractures at room temperature. However, if the tensile temperature is increased to above 523 K, the total elongation can be restored to over 15%, showing an obvious ductile fracture feature [118]. The increased migration rate of point defects and partial dissolution of defect clusters at high temperatures are the main reasons for the recovery of toughness. The tensile properties of W single crystals seriously degrade after neutron irradiation with 0.1–9 dpa [119]. Figure 9 shows that the microstructure of W single crystals severely depends on their irradiation conditions. In the 0.1–0.4 dpa dose range, W single crystals underwent a transition from ductility to brittleness, with a sharp decrease in macroscopic elongation and cross-sectional reduction, consistent with the evolution behavior of dislocation loops and precipitates observed by TEM. At higher doses, the increase in Vickers microhardness reaches 12.9 GPa after 2.8 dpa, which is independent of irradiation temperature or crystal orientation. The significant degradation of mechanical properties above 0.1 dpa is caused by the accumulation of irradiation-induced clusters and the eventual precipitation of transmutation elements Re and Os. Similarly, the irradiation embrittlement behavior of Mo single crystals and polycrystals was compared through three-point bending experiments, and it was found that the ductile–brittle transition temperature (DBTT) of single crystals is much larger than that of the polycrystalline [48]. Recently, Abernethy et al. [49] systematically measured the mechanical properties of W single crystals after fast neutron irradiation with 1.67 dpa at different temperatures and strain rates, and determined that the DBTT increased by about 500 K with increasing irradiation dose. Further analysis shows that the formation of kink pairs controls the brittle–ductile transition behavior of W single crystals, and the irradiation-induced damping of dislocation motion is crucial to the rise of DBTT. In general, irradiation embrittlement behavior is also affected by factors such as material, temperature, and dose. To study its embrittlement mechanism, not only advanced characterization methods are needed, but also the development of multi-scale simulation methods.

To establish the relationship between the initial microstructure, irradiation-induced microstructure, and corresponding irradiation hardening of tungsten single crystals, it was found that the main contribution to hardening at high irradiation temperatures comes from voids and dislocation loops by comparing the irradiation hardening with different microstructures [46]. The indentation test is a common method to evaluate irradiation hardening. However, there is a lack of compression data for tungsten single-crystal indentation model calibration due to most of the existing data coming from tensile tests. Therefore, the multi-scale simulation method is an important means to study the irradiation defects and evolution law. For example, Dellis et al. [120] used the crystal plastic finite element method (CP-FEM) model to simulate the indentation load–depth curve, and the stress distribution was calculated under the indentation to verify the fitting constitutive law. Bonny et al. [121] used the dynamic Monte Carlo simulation tool to simulate the microstructure evolution during neutron irradiation, which improved the rationality of the experimental results. The relationship between radiation microstructure and hardness is explained by the dispersion barrier model. In addition, the crystal plasticity model extended based on the irradiation defect hardening law of discrete dislocation and the defect density evolution law of the molecular dynamic can predict the hardness increase of tungsten single crystals under different irradiation doses, which agrees well with experimental results [122]. The above studies show that irradiation hardening behavior is closely related to material type, irradiation temperature, and irradiation dose.

### 4.4. Effects of Irradiation Damage on Microstructure and Mechanical Properties

Complex defect configurations, such as vacancy clusters, interstitial atom clusters, dislocation loops, helium bubbles, and precipitates, are formed in refractory metal single crystals after irradiation. The nucleation, migration, aggregation, and evolution processes of these defects greatly influence the macroscopic properties of materials. The degradation of material properties is closely related to the irradiation conditions. Singh et al. [123] investigated the effects of neutron irradiation on the microstructural evolution and mechanical properties of single-crystal and polycrystalline molybdenum, as shown in Table 3. It is believed that the production of gliding SIA clusters in displacement cascades plays a decisive role in the evolution of irradiation-induced microstructures (such as the density, size, and distribution of clusters/loops and rafts). The irradiated MoRe single crystals lack raft formation and high-density rings, and the presence of impurity atoms may play a role in reducing the mean free path of the one-dimensional glide of SIA clusters. It is thought that impurity atoms bound to SIAs to a certain extent will interfere with the related motion of SIA clusters that cause one-dimensional slip. In addition, rafts are formed in less pure TZM (compared to MoRe), and the rafts are denser and shorter in length than pure Mo single crystals, which provides qualitative support for the above argument. The decrease in ductility caused by irradiation has been explained by the grain boundary embrittlement caused by separated impurity atoms or due to the inability of the grain interior to plastic deform uniformly. The presence of impurity atoms or alloying elements may influence the gliding process and microstructural evolution.

**Figure 10:**
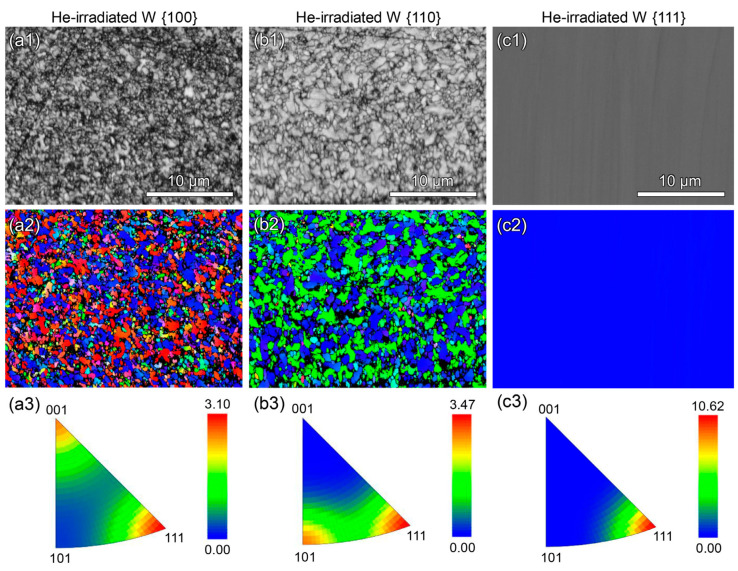
EBSD studies of He-irradiated single crystals [126]: (**a1**–**a3**) W {100}, (**b1**–**b3**) W {110}, and (**c1**–**c3**) W {111}. (**a1**–**c1**): BC maps. (**a2**–**c2**): Normal direction-projected IPF orientation maps; refer to Figure 1 for the unit-triangle color key. (**a3**–**c3**): IPFs.

The detailed behavior of defect evolution can be observed by in situ transmission electron microscopy (TEM) characterization, providing a powerful tool for studying the interaction mechanism of irradiation defects. Yun et al. [124] tracked the microstructural evolution of irradiated Mo single crystals by Xe ions under in situ TEM and found that dislocation loops formed at lower doses and tended to distribute along {111} planes. High-dose irradiation leads to the nucleation and growth of nanoscale helium bubbles, while the He bubbles remain stationary in the Mo matrix, which may be related to the higher diffusion migration energy of defects. Gavish [125] et al. compared the microstructural changes in proton-irradiated W single crystals and polycrystals and found that the nucleation density of helium bubbles in single crystals is much lower than that in polycrystals, and the critical nucleation dose is one order of magnitude higher. This is attributed to the preferential adsorption of helium atoms at grain boundaries, which accelerates the bubble nucleation. In contrast, Fan et al. [126] observed stronger anisotropic effects in irradiated W single crystals by He ions with different crystal planes. After He ion irradiation at room temperature, new surface grains appeared in sets of {100} and {110} planes, while no new grains were in sets of the {111} plane, as shown in Figure 10. Lu et al. [127] believed that the plastic deformation mechanism of the helium bubble is realized by emitting a [100] vacancy-type columnar dislocation loop, which is of the 1/2<111> edge dislocation.

## 5. Conclusions and Prospects

Refractory metal single crystals are indispensable key structural materials for future advanced nuclear reactors due to their unique properties. The preparation technology, deformation behavior, and irradiation damage of refractory metal single crystals are briefly summarized in this review. Overall, both EBFZM and PAM techniques have their respective advantages and disadvantages. However, due to the characteristics of refractory metal single crystals growing under vacuum high-temperature gradients, both techniques have difficulty producing defect-free crystals. Refractory metal single crystals are often subjected to different external loads at corresponding service environments, and the mechanical properties of single crystals including tensile (W), creep (Mo), fatigue (Nb), and impact (Ta) have a strong dependence on temperature and crystal orientation. In addition to the two basic mechanisms of slip and twinning, refractory metal single crystals also exhibit extraordinarily complex deformation behaviors under extreme environments. Importantly, recrystallization is always induced by the combination of deformation and annealing, especially in Nb single crystals (rolling and 1273 K annealing) and Mo single crystals (cold compression and 1473 K annealing), where the destruction of the single-crystal structure is undoubtedly fatal. Refractory metal single crystals suffer complex irradiation damage under fast neutron irradiation, high heat loads, and ion bombardment. Irradiation defects such as vacancies, dislocation loops, and precipitates hinder dislocation motion, causing hardening and neutron embrittlement, which degrade the performance of materials. From the above, important progress has been made in the preparation, deformation, and irradiation research of refractory metal single crystals, greatly advancing the understanding of refractory metal single crystals in the field of advanced nuclear energy. However, there are still some key issues that need to be addressed considering the current state of development, including the following aspects.

**(1)**  **Optimizing the single-crystal growth techniques**

Generally, before single-crystal growth using EBFZM, multiple processes such as electric arc furnace pre-melting are required to prepare polycrystalline billets, resulting in a long production process and increased preparation costs. The PAM method can directly use powder and wire as raw materials to prepare single crystals, reducing or avoiding the preparation of polycrystalline original billets, thereby greatly reducing the processing cost of single crystals [56,62]. However, the PAM equipment structure is complex, with high maintenance costs, and the single crystals prepared by this method have high-density defects. By designing rod materials containing target alloying elements as raw materials, directly preparing low-alloy single crystals with few defects, thereby improving their economy, is the key development direction of future refractory metal single-crystal preparation technology. According to the different types and sizes of refractory metal single crystals, the appropriate preparation technology is selected; in particular, the advantages of EBFZM and PAM are combined to explore the best process and efficiently prepare low-cost and high-quality single crystals.

**(2)**  **Clarifying the deformation mechanism during the machining processing**

The intrinsic brittleness of refractory metal single crystals makes them difficult to deform and process, seriously restricting their development and application. Studying the deformation behavior of refractory metal single crystals can not only enrich the plastic deformation mechanism of BCC metals but also hopefully solve the key problems troubling pressure processing. However, the unique lattice structure of BCC metals creates obstacles to understanding their unique deformation characteristics. Therefore, exploring the root cause and common scientific issues of the ductile–brittle transition in BCC single crystals, establishing the intrinsic correlation between deformation parameters and deformation response, and revealing the complex deformation mechanism of refractory metal single crystals are the primary prerequisites for their plastic processing.

**(3)**  **Characterization and evaluation of the service behavior under extreme environments**

Under extreme environments such as high temperature, strong irradiation, and ultra-long time, it is difficult to carry out physical experiments, posing great challenges to the characterization and evaluation of the service behavior of single crystals. For example, the cost of neutron irradiation experiments above 1073 K is enormous, and available experimental opportunities are strictly limited [128,129]. The combination of basic experiments and simulations is an important research method for studying single-crystal properties and is also an important development direction for characterizing and evaluating the service behavior in extreme environments in the future.

## Figures and Tables

**Table 1:** Development of refractory metal single crystals.

Single-Crystal Types	Preparation Techniques	Dimension (mm)	Applications	References
Mo and W alloy bars	EBFZM	Φ(10~15) × (150~200)	heat-to-current conversion	[50]
Mo and W alloy bars	EBFZM	Φ30 × 600	heat-to-current conversion	[51]
W tubes	EBFZM	Φ(20~30) × 200 × 1		[14]
Mo and W alloy bars	EBFZM	Φ(4~30) × (50~600)		[14]
(100) (110) (111) W plates	EBFZM	7.5 × 7.5 × 2	thermionic dispenser	[52]
[110] Mo-1.5Ta [110] Mo-1.5W [100] Mo-2Re [100] Mo-3Re	EBFZM	—	glass welding material	[53]
Mo-3Re foils	EBFZM	0.5	vacuum seal	[15]
Low-alloyed Mo-Nb; Mo-Hf; Mo-Hf-C bars	EBFZM	Φ15	heat-to-current conversion	[7,9,54]
W bars W tubes W plates W disk	PAM	Φ50 × 300 Φ70 × 10 × *L* 8 × 75 × 160 Φ100	heat-to-current conversion	[55]
[100] W-1.43Mo [110] W-1.62Mo [110] W-1.55Ta [100] Mo-1.81Nb [110] Mo-1.53W [110] Mo-1.48Ta	PAM	—	heat-to-current conversion	[56]
W bars	PAM	—	heat-to-current conversion	[57]

**Table 2:** Tensile and yield strength of monocrystalline and polycrystalline tungsten.

Temperature (°C)	Materials	σ_0.2_ (MPa)	σ_B_ (MPa)	Prolongation (%) or ε_r_	Refs.
25	[001] Pure W	—	760	19.5	[26]
	[001] Pure	—	1040	14.2	[26]
	[001] Pure W	1085	1812	21	[27]
	[001] W-2Re	732	2013	31	[27]
	[001] W-1Mo-1Re	853	1756	24	[27]
1400	W-Poly	79	115	9	[57]
	W-Ta	77	125	13.5	[57]
1800	W-Poly	52.5	73	6	[57]
	W-Ta	52	75	26	[57]
2000	W-Poly	49.3	65.5	4.5	[57]
	W-Ta	43	58	26.5	[57]
2100	W-Poly	45	63	6.5	[57]
	W-Ta	38	49	24	[57]

**Table 3:** Irradiation defect in neutron irradiated monocrystal Mo at 320 K and the tensile test at 295 K.

Materials	Neutron Fluence (n/m^2^ (E > 1 MeV))	Dose (NRT dpa)	Loop Density (10^22^ m^−3^)	Loop Size (mm)	Raft Density (10^22^ m^−3^)	Raft Length (nm)	σ_0.2_ (MPa)	σ_max_ (MPa)	$\varepsilon_{u}^{p}$	ε*_t_*
Mo (Monocrystal)	-	0	-	-	-	-	430	610	2.5	12
	5 × 10^21^	5.4 × 10^−4^	0.33	4.5	-	-	-	-	-	-
	5 × 10^22^	5.4 × 10^−3^	0.82	4.6	0.19	24.2	650	655	1.1	15
	5 × 10^23^	5.4 × 10^−2^	0.77	4.7	0.37	40.9	-	-	-	-
	1.5 × 10^24^	1.6 × 10^−1^	0.76	4.1	0.32	46.1	-	690	0.1	0.6
Mo-5%Re (Monocrystal)	-	0	-	-	-	-	375	500	2.5	9.0
	-	5.4 × 10^−4^	-	-	-	-	445	502	2.5	8.5
	5 × 10^22^	5.4 × 10^−3^	12.0	1.3	-	-	-	-	-	-
	5 × 10^23^	5.4 × 10^−2^	13.1	3.0	-	-	625	700	0.3	7.0
	1.5 × 10^24^	1.6 × 10^−1^	5.2	5.6	-	-	-	1275	1.3	2.5

## Data Availability

Not applicable.
